# Early experience with total robotic D2 gastrectomy in a low incidence region: surgical perspectives

**DOI:** 10.1186/s12893-022-01576-1

**Published:** 2022-04-09

**Authors:** Tom Mala, Dag Førland, Caroline Skagemo, Tom Glomsaker, Hans Olaf Johannessen, Egil Johnson

**Affiliations:** grid.55325.34 0000 0004 0389 8485 Dep. of Gastrointestinal and Pediatric Surgery, Division of Surgery, Inflammatory Medicine and Transplantation, Oslo University Hospital, Ullevål Hospital, Nydalen, Pb. 4956, 0424 Oslo, Norway

**Keywords:** Gastrectomy, Cancer, Outcome, Feasibility, Minimal invasive, Oncology, Robotic surgery, Gastric cancer, Surgery

## Abstract

**Background:**

Few European centers have reported on robotic gastrectomy for malignancy. We report our early experience with curative-intent total robotic gastrectomy.

**Materials and methods:**

The Intuitive Surgery Da Vinci Surgical System Xi 4 armed robot was used. Routine D2 lymphadenectomy was applied.

**Results:**

Some 27 patients with adenocarcinoma (n = 18), hereditary cancer susceptibility (n = 8) and premalignancy (n = 1) were allocated to robotic gastrectomy, three were excluded due to inoperability during surgery. Median (range) age was 66 (18–87) years, 14 (58.3%) were females and body mass index was 25.5 (22.1–33.5) kg/m^2^. Total gastrectomy was performed in 19 (79.2%) and subtotal in five (20.8%) patients. One (4.2%) procedure was converted to laparotomy. Procedural time was 273 (195–427) minutes. Three (12.5%) patients were reoperated within 30 days, one (4.2%) died. Serious complications (Clavien Dindo IIIb or more) occurred in three (12.5%) patients. Postoperative hospital stay was 10 (6–43) days. Fourteen of 16 (87.5%) patients with adenocarcinoma/premalignancy received radical resections. The median number of harvested lymph nodes was 20 (11–34). Eleven (73.3%) patients with adenocarcinoma had T3/T4 tumors and 6 (40%) had TNM stage III or more.

**Conclusion:**

Total robotic D2 gastrectomy appears feasible and safe during early introduction in a low incidence region.

## Background

Gastrectomy for cancer is widely performed by minimally invasive surgery. In a meta-analysis laparoscopic gastrectomy was reported not inferior to open surgery in regard to short and long-term oncologic outcome. Measures of early postoperative recovery and rate of total complications were in favor of laparoscopy [1].

Evidence from a wide range of series, most Asian, indicates that minimally invasive robotic gastrectomy may also be performed with short-term oncologic outcome comparable to that of open surgery [2, 3]. Available reports include series of robotic assisted and total robotic surgery for distal and total gastrectomy [4–6]. A recent randomized controlled trial reported benefits of robotic compared to laparoscopic distal gastrectomy in regard to morbidity, recovery, and lymphadenectomy [7]. In a meta-analysis pooled data from retrospective comparative studies support these findings [8]. Robust evidence, however, for the widespread use of robotic gastrectomy is pending [5].

The paucity of data, particularly from European centers, underlines a need for rigorous assessment of efficacy and outcome [9]. Experiences across regions, cohorts, and types of gastric resection, remain important contributions to clinical robotic protocols until evidence comes to maturity. A 2019 review of European reports of robotic versus open or laparoscopic surgery included 123 robotic procedures. Compared to Asian series the patients had higher body mass index, more advanced tumour stage, and total gastrectomies were more common [2]. To our knowledge no Scandinavian series of robotic gastrectomy for malignancies have been reported.

We report our early experience with curative-intent robotic gastrectomy in a Norwegian cohort focusing on safety, technical aspects, and oncologic short-term outcome.

## Methods

Oslo University Hospital is a referral institution for curative-intent treatment of gastric cancer in South East Norway (population about 2.9 millions). Annual regional number of resections is some 50 patients, including about 30 at Oslo University Hospital. Selected patients from other regions such as patients with Hereditary Gastric Adenocarcinoma and Proximal Polyposis of the Stomach (GAPPS) are also refered to our hospital. Neoadjuvant chemotherapy, primarily by the FLOT 4 regime, was part of multimodality treatment in patients < 75 years with acceptable comorbidity.

Patients referred for cancer, GAPPS, and precancerous lesions were considered for robotic gastrectomy. Laparoscopy has been the institutional routine approach to gastrectomy since 2015 [10]. Allocation to robotic resection was based on the availability of two surgeons performing robotic surgery, slot time for use of the robot, and no significant delay of scheduled treatment. The two surgeons were experienced in laparoscopic gastrectomy and had completed a certification process for robotic surgery. The assisting surgical nurses were experienced in robotic surgery from other procedures. The first procedure was performed November 2018 and tutored by a surgeon proficient in robotic gastrectomy for malignancy. The last patient was included by December 2020. Patients were admitted to the hospital the day before surgery and received prophylactic low molecular heparin and antibiotics. Epidural analgesia was established.

### Surgical technique

**Fig. 1:**
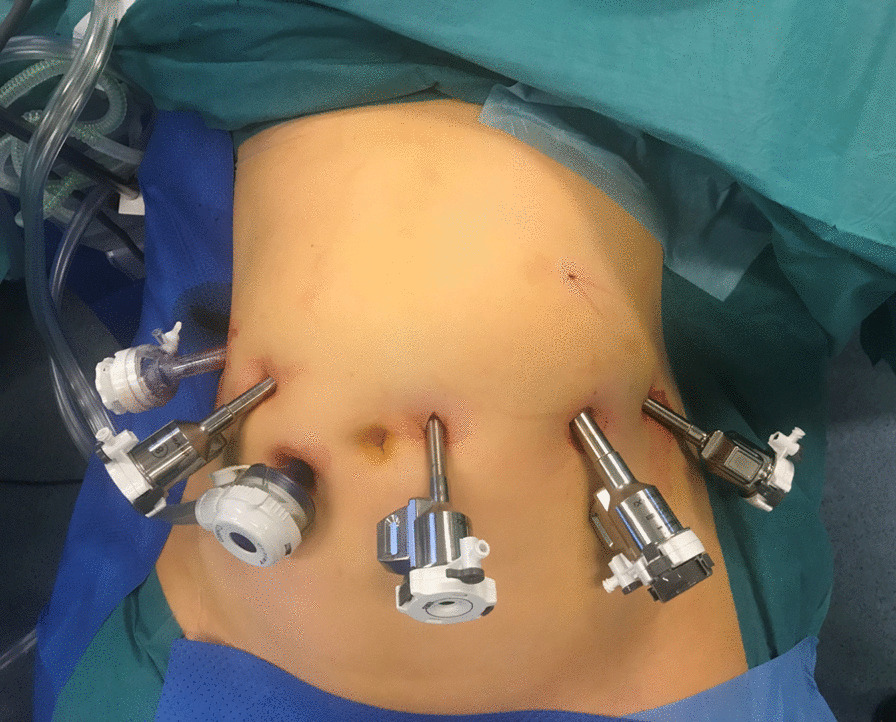
Trocar placement during robotic total and subtotal D2 gastrectomy. Three 8 mm robotic trocars and one 12 mm robotic trocar were used and positioned at the level of the umbilicus. In addition a 5/12 mm port (liver retraction) in the right hypochondrium, and a 12 mm assistant trocar for suction, clipping and introducing/removing sutures were used

The patients were positioned in an anti Trendelenburg (14°) supine position, and the table was rotated 4° to the patients right side. The Intuitive Surgery Da Vinci Surgical System Xi 4 armed robot was positioned at the left side of the patient. Verres needle was used to establish pneumoperitoneum. Three robotic 8 mm and one robotic 12 mm ports were used separated by 7 cm at the umbilical level. Two additional trocars for liver retraction (5/12 mm) and manual assistance (12 mm), respectively, were applied (Fig. 1).

**Fig. 2:**
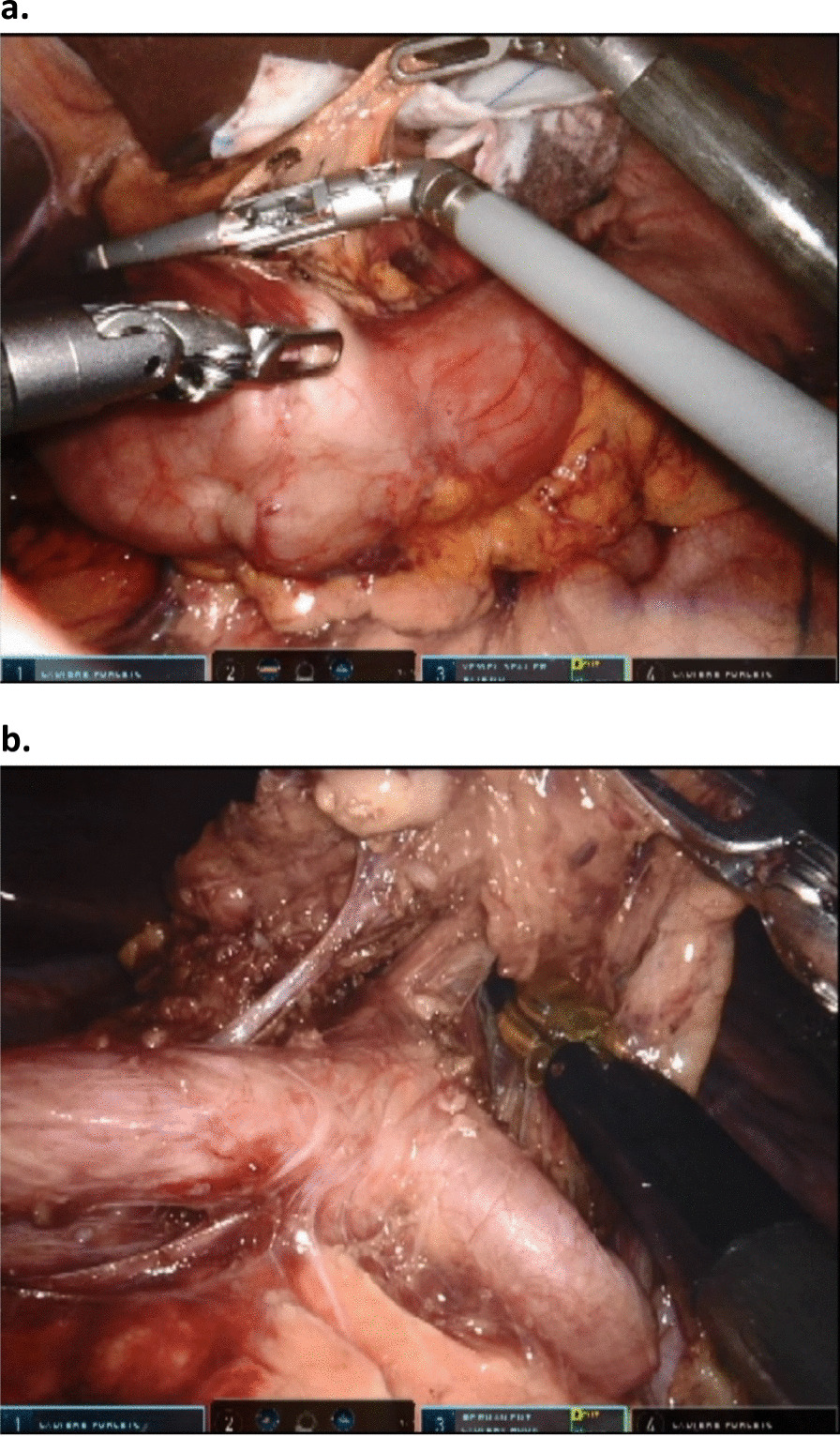
Use of the Vessel Sealer during robotic mobilization of the antrum (**a**). The three robotic arms and the camera arm are all in use. **b** Depicts the use of the electric hook for detailed dissection of the hepatic and the splenic artery. The artery pointing upwards is the left gastric artery

A modified D2 resection was performed in all patients including the patients receiving prophylactic gastrectomy for GAPPS. Lymphadenectomy included stations 1, 2, 3, 4, 5, 6, 7, 8, 9 and 11 and 12 in total gastrectomy. Station 2 and parts of 4 were not resected during subtotal gastrectomy to preserve circulation to the gastric remnant. This approach is in line with national guidelines and our institutional routine [10]. An electric robotic hook was used for delicate lymph node dissection, otherwisee the robotic Vessel Sealer was used (Fig. 2).The duodenum was divided using a linear 60 mm blue robotic cartridge. Indocyanine fluorescent dye with infrared imaging was not applied.

**Fig. 3:**
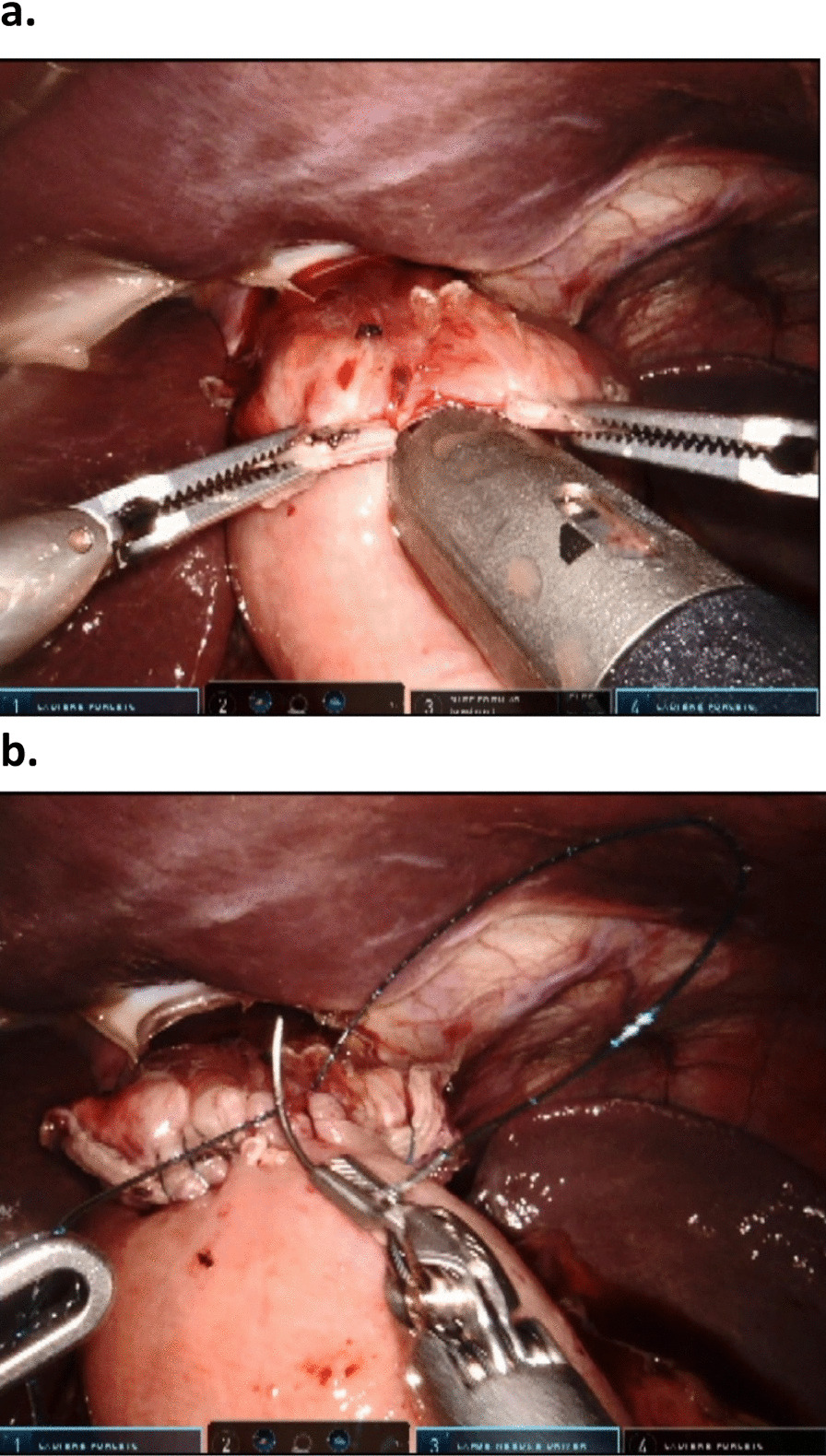
Establishment of the esophagojejunostomy during robotic total gastrectomy. In **a** the linear stapler is in place to establish the anastomosis, and in **b** the final anastomosis following robotic suturing is shown

In total gastrectomy the esophagus was divided using a linear robotic 60 mm green cartridge. The distal end of the esophagus was mobilized and the main branches of the vagal nerve were divided. The ligament of Treitz was identified and about 40–50 cm more distal a loop of the jejunum was sutured to the left or the right diafragmal crura. About 60–70 cm distal to the suture a side to side jejunojejunostomy was made with the loop just proximal to the suture using a 45 or 60 mm robotic stapler blue cartridge. The intestinal defect after removal of the stapler was closed by robotic suturing using absorbable V-Lock 3-0 suture. The small intestine was subsequently divided between the jejunojejunostomy and the crural suture (60 mm blue cartridge) thus creating a blind ended alimentary limb. The jejunal mesenterium at the enteroenteroanastomosis was split centrally a few cm to allow for fall down of the anastomosis. These steps compare well to the widely adopted loop technique for bariatric gastric bypass. Subsequently, the esophagojejunal anastomosis was established by an proximal enterotomy at the blind ended alimentary limb using a linear stapler 45 mm green cartridge (Fig. 3). The intestinal defect was closed using absorbable V-lock 3-0 sutures from both ends of the opening.

In subtotal gastrectomy the stomach was divided horizontally at the appropriate site using 2–3 60 mm blue cartridges. A loop 40–50 cm distal to the ligament of Treitz was then used to make the gastrojejunostomy centrally situated on the gastric remnant staple line (45 mm robotic linear blue cartridge). The opening after removal of the stapler was closed using absorbable V-Lock 3-0 from both ends of the opening. About 60–70 cm distal to the anastomosis a side to side jejunojejunostomy was made (60 mm or 45 mm blue cartridge) closing the instrumental intestinal defect using an absorbable V-lock 3-0 suture. The jejunum loop was divided (blue cartridge 60 mm) between the jejunojejunostomy and the gastric remnant completing the Roux-en-Y reconstruction with a 60–70 cm alimentary limb and about 40-50 cm biliopancreatic limb. An antecolic route was used for the alimentary limb. All stapling was performed using robotic stapling devices for both total and subtotal gastrectomy.

The upper anastomosis was tested with methylene blue for leakage and run-off. A vacuum drain was positioned close to the anastomosis. Specimens were retrieved trough a minor extension of one of the trocars or trough a suprapubic incision. The patients started drinking the same day limiting oral intake to fluids for 7 days when food was gradually introduced. Drains were typically removed day 3–4 following amylase measurements of drain fluids.

### Definitions and ethics

Serious complications were graded according to the Clavien-Dindo classification system (IIIb or more) [11]. Perioperative complications and death were defined as within 30 days. Surgical time indicates the time period from insertion of Verres needle until last skin sutures were set. Docking time was defined as the time interval between insertion of the Verres needle and start of dissection.

All patients were informed and consented to the use of robotic surgery. The use of patient data was approved by the local data protection officer at Oslo University Hospital according to institutional and national guidelines and the study was classified as a quality assurance study. The need for ethical approval was waived in agreement with the Regional Ethical Committee (Health Region South East Norway- Committee D) according to national regulations (Health Research Act § 2 and § 4 letter a). Written approval for use of pictures was retrieved from the patients.

## Results

A total of 27 out of 77 (35.1%) patients operated for gastric cancer, susceptibility for hereditary cancer or premalignancy were allocated to robotic gastrectomy. Patient characteristics are given in Table 1.

**Table 1:** Preoperative patient characteristics of 24 patients operated with robotic D2 gastrectomy at Oslo University Hospital

Patient characteristics	
Females/males, no	14/10
Age, years (median/range)	66 (18–87)
ASA, No	
1	5
2	14
3	5
BMI, kg/m^2^ (median/range)	25.5 (22.1–33.5)
Adenocarcinoma, no	
GAPPS, no	15
High grade dysplasia, cancer uncertain, no	8
Preoperative T (tumor) stage^a^, no	1
Tx	1
T1	1
T2	4
T3	7
T4	2
Preoperative N stage^a^, no	
Nx	1
N0	7
N1	5
N2	2
Preoperative chemotherapy, no	9

Three additional patients were inoperable due to advanced disases at time of surgery and are not included

*No* number of patients, *GAPPS* gastric adenocarcinoma and proximal polyposis of the stomach, *BMI* body mass index, *x* undefined

^a^For 15 patients with adenocarcinoma,

Three (11.1%) patients were deemed inoperable due to metastatic or locally advanced disease, two following robotic release and mobilization of the stomach, one immediately after introducing the optic trocar. These were excluded from further analyses.

**Fig. 4:**
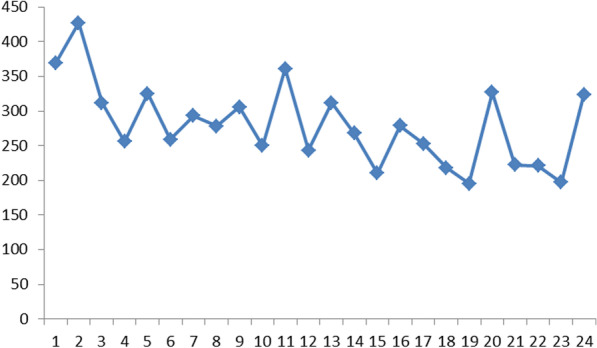
Procedural time (minutes) trajectory for 24 conseutive robotic total (n = 19) and subtotal (n = 5) D2 gastrectomies at Oslo University Hospital

Of the remaining 24 patients total and subtotal robotic gastrectomy was performed in 19 (79.2%) and 5 (20.8%) patients, respectively. Duration of surgery was median 273 (range 195-427) minutes, docking time for patients during the last part of the period was 18 (16-22) minutes. Total procedural time for the individual consecutive procedures is given in Fig. 4. Estimated intraoperative blood loss was less than 150–200 ml in 22 patients and less than 200–300 ml in 2 patients. One (4.2%) patient was converted to laparotomy due to jejunal tension during establishing of the esophagojejunostomy. All other steps of the procedure including the jejunojejunostomy were completed robotically.

Postoperative complications afflicting 12 (50%) patients are listed in Table 2. Three (12.5%) patients were reoperated. One for anastomotic leak at the gastrojejunostomy (subtotal resection), one for undefined bleeding probably related to abdominal wall, and one from adhsions after previous appendectomy causing intestinal obstruction. The patient with ischemic findings of the mucosa in the upper anastomosis by gastroscopy was handled with nil per os and gradual reintroduction of oral fluids after 3 days with no complications related to this. One (4.2%) patient died from an anastomotic leak. Hospital stay was 10 (6–43) days.

**Table 2.** Perioperative complications (within 30 days) of 24 patients operated with robotic D2 gastrectomy at Oslo University Hospital

Complications	No. (%)
Patients with one/more complications (%)	12 (50)
Reoperation (bleeding, anastomotic leak, ileus)	3 (13)
Pneumonia	4 (17)
Urinary tract infection	3 (13)
Pleural drainage	2 (8)
Intraabdominal abscess^a^	2 (8)
Hospital readmission (30 days)	1 (4)
Wound infection	1 (4)
Peripheral pulmonary embolism	1 (4)
Unspecified CRP elevation (antibiotics)	1 (4)
Nutritional problems prolonging stay	1 (4)
Transient drop foot (peroneal nerve compression)	1 (4)
Ischemia at esophagojejunostomy	1 (4)
Anastomotic leakage	1 (4)
Death	1 (4)
Patients with serious complications^b^	3 (13%)

*No* number, *CRP* C-reactive protein

^a^One handled with antibiotics, one with drainage and antibiotics. For both patients CT (contrast) and ^gastroscopy without leakage^

^b^Clavien Dindo IIIb or more

Specimen findings are listed in Table 3. None of the patients with GAPPS had malignancy identified. For the remaining 16 patients 12 (75%) had 16 or more lymph nodes examined. The resection margins (proximal, distal and circumferential) were free from tumour tissue in 14 (87.5%) of these patients. In two (12.5%) patients the resection margin status was uncertain. Eleven (73.3%) patients with adenocarcinoma had T3/T4 tumors and 6 (40%) had TNM stage III or more tumours.

**Table 3:** Specimen findings from 24 patients operated by robotic total (n = 19) and subtotal (n = 5) D2 gastrectomy at Oslo University Hospital

Specimen findings	
Number of lymph nodes, No. (median/range)^a^	20 (11–34)
Uncertain radical resection margin, No.^b^	
Tumor size, mm (median/range)^b^	2
T (tumor) stage, no.^b^	52 (15–100)
T1	3
T2	1
T3	10
T4	1
N (nodal) stage, no. ^b^	
N0	7
N1	1
N2	5
N3	2
Adenocarcinoma, no	15
GAPPS, no	8
High grade dysplasia, no	1

*No* number of patients, *GAPSS* hereditary gastric adenocarcinoma and proximal polyposis of the stomach

^a^In 3 patients with GAPSS, lymph node status was not evaluated

^b^Patients with adenocarcinoma

## Discussion

This study is among few series reporting total robotic approach to gastrectomy for cancer in Europe. Our results show that robotic D2 gastrectomy in a low incidence region is feasible and can be done in experienced hands. Most patients had advanced tumor stage and most received total gastrectomy. Our findings compares at large to our outcome of laparoscopic D2 resections [10]. Benefits of robotics include 3D vision, camera stability, improved instrumental movement with endo-wristed instrumentation, tremor filtration, and improved ergonomics.

Circular stapling is our routine for the esophagojejunostomy during laparoscopic total gastrectomy but may be more time consuming during robotic surgery. We applied linear stapling for this anastomosis making the procedure entirely robotic. According to literature, anastomotic strictures may be less frequent using the linear stapler [12]. The linear anastomosis, however, extends several centimeters up the esophagus contrary to the circular stapled anastomosis located at or near the the end of the esophagus. The blind ended part of the alimentary limb probably benefits from being short. We later (2 months after surgery) shortened this blind end in one patient and repositioned herniated jejunum from the mediastinum due to symptoms of obstruction.

Intestinal perforation from instrumenting is a risk particularly prior to proficiency. Tension when mobilizing the intestines to establish the esophagojejunostomy was the cause of laparotomy in one patient. Perforation by the stapling device of the alimentary limb during this step occurred in two additional patients, but was immediately handled. Magnification during robotic surgery may contribute to creation of shorter intestinal limb lengths compared to laparoscopic surgery, and we currently use a 10 cm band to estimate limb lengths.

We used the Vessel Sealer and the electric hook during lymph node dissection. Although providing excellent hemostasis we find the Vessel Sealer plump for delicate dissection and refinements would probably facilitate dissection. Indocyanine fluorescent dye with infrared imaging may be used during robotic surgery to assess vascularity and to identify lymph nodes. This was not applied in our series but has been shown to facilitate lymph node dissection during laparoscopic gastrectomy [13].

Robotic gastrectomy is consistently reported more time consuming than laparoscopic resection. Although the number of instrumental exchanges during surgery may be comparable to laparoscopy, the time needed for each change is longer during robotic surgery [14]. Instruments with multiple applications may reduce time consumption. The Xi system is designed for multi quadrant access reducing the need for re-docking. Our operative time of 273 min compares to the 265 min for our first series of laparoscopic D2 gastrectomies [10]. We observed a trend towards reduced operation time during the period (Fig. 2). Docking time was by the end of the series relatively stable about 18 minutes. Proficiency in robotic gastrectomy has been reported to 20–30 cases and mastery by 60–80 cases. The learning curve may be shorter compared to laparoscopic gastrectomy which could be an advantage particularly in low volume centers [3, 9, 15].

All but one procedure was completed robotically. Risk factors for conversion to laparotomy include higher body mass index, larger tumor size and neo-adjuvant chemotherapy. Patients with several of these factors may be at particularly high risk [3]. Studies report comparable morbidity after robotic and laparoscopic gastrectomy. However, reduced blood loss is commonly reported after robotic resection, and some report reduced risk of pancreatic fistula formation [2, 8, 9]. Our complication rates were comparable to our laparoscopic experience; total morbidity was 50% after robotic resection and 52% after laparoscopic gastrectomy. The corresponding figures for serious complications were 12.5% and 12%, reoperation rates were 12.5% and 9%, and mortality rates were 4.2% and 3%, respectively. Notably, total gastrectomy comprised most of our robotic resections (79.2%) adding complexity and risk of morbidity compared to subtotal resections. The 10 days hospital stay after robotic resection also compares to the 12 days hospital stay after laparoscopic gastrectomy [10]. The small sample size, however, prevents rigorous conclusions to be made. The relative long hospital stay may relate to high age in some of the patients, long travel distances to the hospital and initial challenges with adequate nutritional intake.

The oncological short-term safety of robotic gastrectomy is reported comparable to open and probably laparoscopic resection [4–6, 16]. A recent randomized controlled trial of robotic versus laparoscopic gastrectomy showed improved lymphadenectomy by the robotic approach for extraperigastric lymph nodes including station 12a. This suggests improved compliance to D2 lymphadenectomy in the robotic arm [7]. If confirmed this may translate into improved long-term outcome. Our oncologic findings are in line with our laparoscopic experience to gastrectomy for cancer [10]. In our robotic series resectional radicality was uncertain/not radical in two (13.3%) patients with adenocarcinoma : one with <1 mm circumferential margin in a T3 tumour with diffuse growth, and one with uncertain distal margin for a 9 cm antral T3 tumour. In our laparoscopic gastrectomy experience 8% of the patients had involved or uncertain resection margin status. The number of lymph nodes sampled was median 20 nodes in the present robotic series compared to 18 in our laparoscopic series [10]. In our experience, the robotic approach may have special advantages during gastrectomy compared to laparscopy when operating in narrow spaces like when suturing the esophagojejunal anastomosis. We found the 3D and steady camera view beneficial during lymph node dissection and combined with the surgeons self-control with all the four robotic arms this may facilitate lymph node dissection. However, the robotic approach did not extend indications for surgery or resectability as compared to the laparoscopic approach.

The current cost-effectiveness of robotic gastrectomy is reported inferior to that of the laparoscopic approach [15]. This disadvantage may be reduced with technical improvements and substantiated experience with robotic systems. The increased use of robotic systems itself may be drivers in this regard.

GAPPS is a rare entity without rigorous treatment guidelines. We support a standard D2 gastrectomy in patients with a family history, typical endoscopic findings and verified mutations in the adenomatous polyposis coli (APC) promoter genes as early cancer may be overlooked by endoscopic surveillance [17, 18]. However, evidence for the extent of lymphadenectomy is not established for these patients. None of our patients had malignancy identified in resected gastric specimens or in regional lymph nodes.

More than 100 patients were operated with D2 gastrectomy laparoscopicaly at the institution prior to start of the robotic program. Moving direct from open to robotic surgery may pose challenges not evaluated in our study. Although selection biases may be present as about 1/3 of eligible patients only were allocated to robotic resection, we believe the patients with cancer were relative representative of our general gastric cancer cohort. The restricted sample size and the single center design are limitations of the present study.

## Conclusions

Total robotic D2 gastrectomy appears feasible in selected patients during the implementation of a robotic gastrectomy program. However, continuous scrutiny is required to ensure robust evidence for the use and potential benefits for patients with malignant disease.

## Data Availability

Due to Norwegian legislation patient data may not be freely availble: in this series there are thus limited availability due to small series and anonymity. Aggregated anonymous data can be available by contacting the corresponding author.

## Abbreviations

GAPPS: Hereditary gastric adenocarcinoma and proximal polyposis of the stomach
APC: Adenomatous polyposis coli
